# Deep-learning automated quantification of longitudinal OCT scans demonstrates reduced RPE loss rate, preservation of intact macular area and predictive value of isolated photoreceptor degeneration in geographic atrophy patients receiving C3 inhibition treatment

**DOI:** 10.1136/bjo-2022-322672

**Published:** 2023-04-24

**Authors:** Dun Jack Fu, Sophie Glinton, Veronika Lipkova, Livia Faes, Bart Liefers, Gongyu Zhang, Nikolas Pontikos, Alex McKeown, Lukas Scheibler, Praveen J Patel, Pearse A Keane, Konstantinos Balaskas

**Affiliations:** 1 NIHR Biomedical Research Centre, Moorfields Eye Hospital NHS Foundation Trust & UCL, Institute of Ophthalmology, London, UK; 2 Department of Ophthalmology, Erasmus University Medical Center, Rotterdam, The Netherlands; 3 Apellis Pharmaceuticals Inc, Waltham, Massachusetts, USA; 4 University College London, London, UK

**Keywords:** Clinical Trial, Imaging, Macula, Retina

## Abstract

**Objective:**

To evaluate the role of automated optical coherence tomography (OCT) segmentation, using a validated deep-learning model, for assessing the effect of C3 inhibition on the area of geographic atrophy (GA); the constituent features of GA on OCT (photoreceptor degeneration (PRD), retinal pigment epithelium (RPE) loss and hypertransmission); and the area of unaffected healthy macula.

To identify OCT predictive biomarkers for GA growth.

**Methods:**

Post hoc analysis of the FILLY trial using a deep-learning model for spectral domain OCT (SD-OCT) autosegmentation. 246 patients were randomised 1:1:1 into pegcetacoplan monthly (PM), pegcetacoplan every other month (PEOM) and sham treatment (pooled) for 12 months of treatment and 6 months of therapy-free monitoring. Only participants with Heidelberg SD-OCT were included (n=197, single eye per participant).

The primary efficacy endpoint was the square root transformed change in area of GA as complete RPE and outer retinal atrophy (cRORA) in each treatment arm at 12 months, with secondary endpoints including RPE loss, hypertransmission, PRD and intact macular area.

**Results:**

Eyes treated PM showed significantly slower mean change of cRORA progression at 12 and 18 months (0.151 and 0.277 mm, p=0.0039; 0.251 and 0.396 mm, p=0.039, respectively) and RPE loss (0.147 and 0.287 mm, p=0.0008; 0.242 and 0.410 mm, p=0.00809). PEOM showed significantly slower mean change of RPE loss compared with sham at 12 months (p=0.0313). Intact macular areas were preserved in PM compared with sham at 12 and 18 months (p=0.0095 and p=0.044). PRD in isolation and intact macula areas was predictive of reduced cRORA growth at 12 months (coefficient 0.0195, p=0.01 and 0.00752, p=0.02, respectively)

**Conclusion:**

The OCT evidence suggests that pegcetacoplan slows progression of cRORA overall and RPE loss specifically while protecting the remaining photoreceptors and slowing the progression of healthy retina to iRORA.

**WHAT IS ALREADY KNOWN ON THIS TOPIC**

- Intravitreal C3 inhibition has been shown to reduce the growth rate of geographic atrophy (GA) in the FILLY phase 2 randomised controlled trial based on manual grading of fundus autofluorescence imaging. Previous work applying automated optical coherence tomography (OCT) segmentation of the FILLY study dataset indicated a predictive role for the ratio of photoreceptor degeneration (PRD) to retinal pigment epithelium (RPE) loss for predicting GA area growth.

**WHAT THIS STUDY ADDS**

- This study confirms the statistically significant effect of C3 inhibition on GA growth rate reduction based on previously validated fully automated deep-learning segmentation of OCT imaging, which uniquely applies the Classification of Atrophy Meeting Consensus definitions for GA. Additionally, it demonstrates the effect of C3 inhibition on rate reduction of RPE loss, as well as on longer preservation of intact macular areas. Finally, it reveals the predictive value of intact macular areas and PRD in isolation, for reduced risk of GA growth, providing the pathophysiological underpinning of previously reported findings.

**HOW THIS STUDY MIGHT AFFECT RESEARCH, PRACTICE OR POLICY**

- This study highlights the potential of automated deep-learning OCT analysis to enable fast and reliable point-of-care monitoring of GA progression and identifies novel OCT imaging features predictive of GA growth, already from the earliest stages of the disease.

## Introduction

Geographic atrophy (GA) is the progressive, irreversible form of late non-neovascular age-related macular degeneration (AMD).^1^ It is characterised by degeneration of the outer retinal layers, retinal pigment epithelium (RPE) and choriocapillaris, and Müller cell gliosis resulting in impairment of vision and quality of life.^2^ The public health burden of GA is significant with more than 5 million people affected globally and is projected to increase with an ageing population.^1^ Although its progression is gradual—thus providing a therapeutic window of opportunity—effective treatments for GA have been elusive, despite intense efforts for therapeutic discovery.^3^

Recently, the clinical trial programme of intravitreal pegcetacoplan, a complement component 3 (C3) and complement 3b (C3b) inhibitor, consisting of the phase 2 (FILLY, ClinicalTrials.gov ID NCT02503332) and the phase 3 trials (OAKS and DERBY, ClinicalTrials.gov NCT03525613 and NCT03525600, respectively), has reported dose-dependent reductions in GA lesion growth rates and demonstrated a favourable safety profile.^4–6^ The primary endpoints of each trial measured change in lesion size of GA at 12 months with manual segmentation of fundus autofluorescence imaging (FAF).

Historically, FAF enabled much of GA research as the licensing-level standard of imaging.^4^ However, there are limitations to using FAF, namely its inability to scrutinise the morphological changes across individual retinal layers or detect the disease in precursor stages.^5 6^ For example, the topography of autofluorescence is tightly linked to the photoreceptor distribution.^7 8^ In addition to screening by photoreceptors, FAF signal is also impacted by rounding, stacking and anterior migration of individual RPE cells.^9^ In response, the international Classification of Atrophy Meeting (CAM) group put forth spectral domain optical coherence tomography (SD-OCT) as the emerging reference imaging standard to describe GA, as it captures the morphology of neuroretinal layers, RPE and choroid in three dimensions while being simultaneously ubiquitous in the clinical environment and rapid, comfortable and non-invasive for the patient.^10^ In contrast to the 3D nature of SD-OCT, FAF signal is understood to arise specifically from lipofuscin accumulation in RPE cells.^11^ There is ambiguity in the literature regarding what this FAF signal represents pathologically in the context of GA histology and retinal cross-sections.^10 12^ For example, FAF hypoautofluorescence can correspond to RPE loss, as would be expected in GA; however, it can also reflect the variable integrity of photoreceptors, complicating signal interpretation.^12 13^ The CAM group leveraged the advantages of SD-OCT to provide consensus definitions of macular atrophy in the context of AMD based on the affected histological correlates as identified by SD-OCT, termed complete RPE and outer retinal atrophy (RORA), and these have since been clinically validated.^14^ Consequently, SD-OCT features have been put forth as future trial endpoints for GA by the National Eye Institute and Food and Drug Administration.^4^

Reporting of GA studies using the CAM-defined OCT endpoints is challenging as such studies require manual segmentation, which is time-consuming, labour-intensive and can feature inter-rater variability.^15 16^ Fortunately, GA research using CAM-defined OCT features to detect and quantify each of the GA subtypes by means of a recently developed deep-learning based platform is not subject to such limitations and has demonstrated performance comparable to human specialist graders on an independent validation set.^17 18^

This post hoc analysis uses the afore-mentioned analytical platform to report the effect of intravitreal pegcetacoplan on the area of cRORA and each of its CAM-defined constituent features (RPE loss, photoreceptor degeneration (PRD) and hypertransmission) at the OCT level. PRD is defined according to the CAM consensus as evidence of impairment of one or more of the following outer retinal layers: outer limiting membrane loss, ellipsoid zone loss, interdigitation zone loss or outer nuclear layer thinning. Furthermore, pegcetacoplan’s effect on macular areas free of GA changes (ie, ‘intact macula’) and on isolated PRD is also reported, capturing what has been proposed as the earliest morphological OCT change in GA.^19 20^ We also consider the topographical location of cRORA features, thereby quantifying the effect of pegcetacoplan on lesion perimeter, focality and position relative to the fovea. Finally, we investigate PRD in isolation and ‘intact macula’ as predictors of iRORA/cRORA development or growth and, therefore, as potential important clinical endpoints for therapy development and disease monitoring.

## Methods

### Study design and cohort

**Figure 1:**
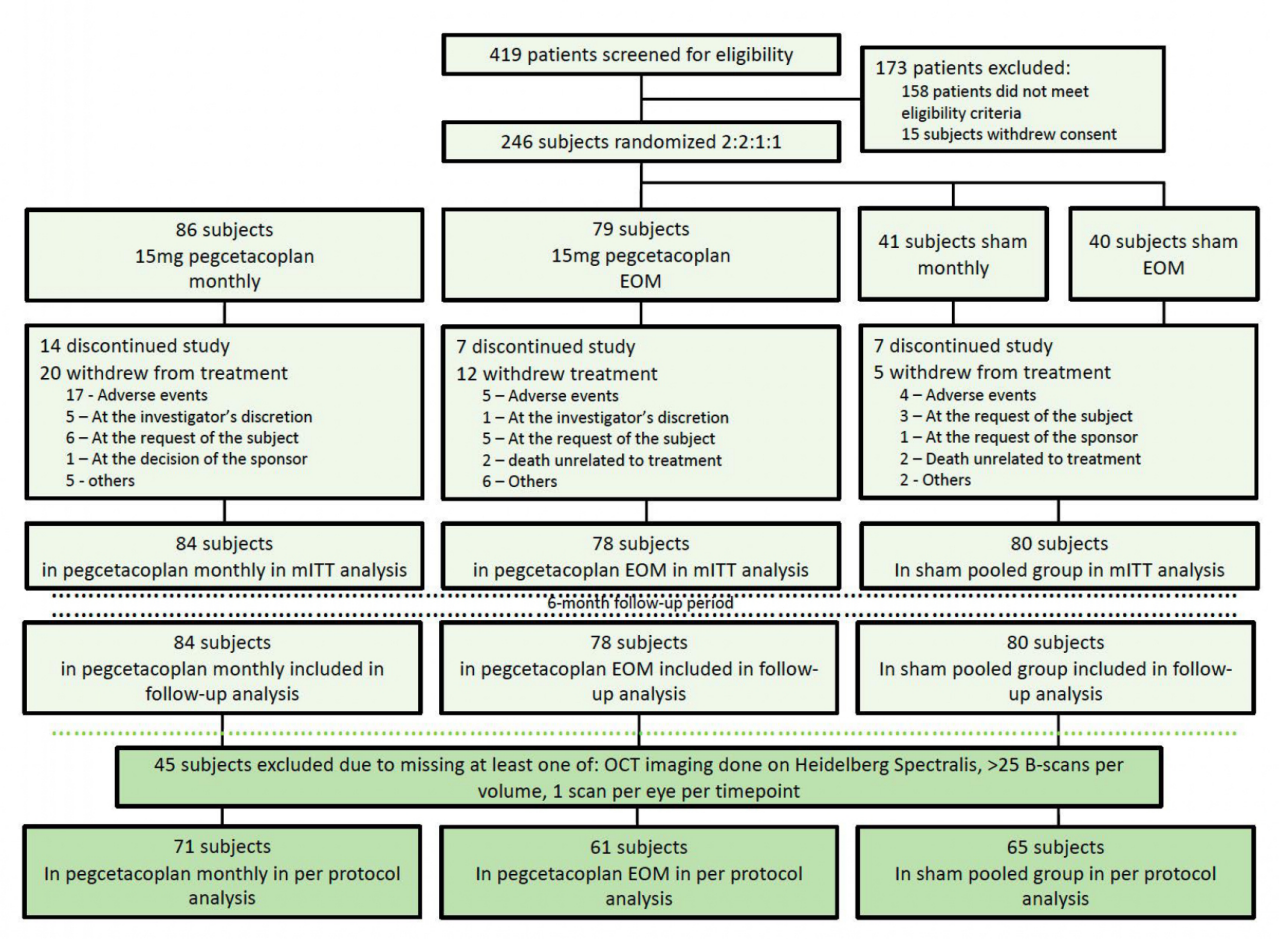
Consolidated Standards of Reporting Trials (CONSORT) flow diagram. A total of 419 patients were screened for eligibility in the FILLY trial. Those who did not consent or did not meet eligibility criteria were excluded from the study, and the remaining 246 subjects were randomised 2:2:1:1 to pegcetacoplan monthly, pegcetacoplan every other month (EOM), sham treatment monthly and sham treatment EOM. During the follow-up period, 65 subjects left the trial and the remainder were followed up for a total of 18 months (12 months of treatment and 6 months of follow-up monitoring). The optical coherence tomography (OCT) analysis (dark green) excluded patients who did not meet the following criteria: OCT imaging done on Heidelberg Spectralis, OCT with less than 25 B scans per volume, at least 1 scan per eye per time point. The remaining subjects were analysed per protocol. Reproduced from the FILLY trial CONSORT diagram. mITT, modified intention to treat.

This is a post hoc analysis of participants enrolled in the FILLY trial (NCT02503332)—a prospective, multicentre, sham-controlled, randomised phase 2 trial evaluating the safety and efficacy of intravitreal pegcetacoplan in eyes with GA secondary to AMD (figure 1).^5^ Patients stopped receiving injections at month 12 but continued to be followed up for a further 6 months. All participants with OCT volumes acquired using Heidelberg Spectralis OCT and HRA (Heidelberg Engineering, Heidelberg, Germany) and with 25 or more B scans covering 6×6×2 mm^3^ were selected. A total of 92 097 B scans of 1876 volumes of 197 eyes were taken forward for analysis; using a single study eye per patient, 71 were randomised to pegcetacoplan monthly (PM), 61 to pegcetacoplan every other month (PEOM) and 65 to sham pooled groups. In cases of multiple OCT volume scans for a given eye at a given time point, the scan with the highest number of B scans was used.

### Image analysis workflow

**Figure 2:**
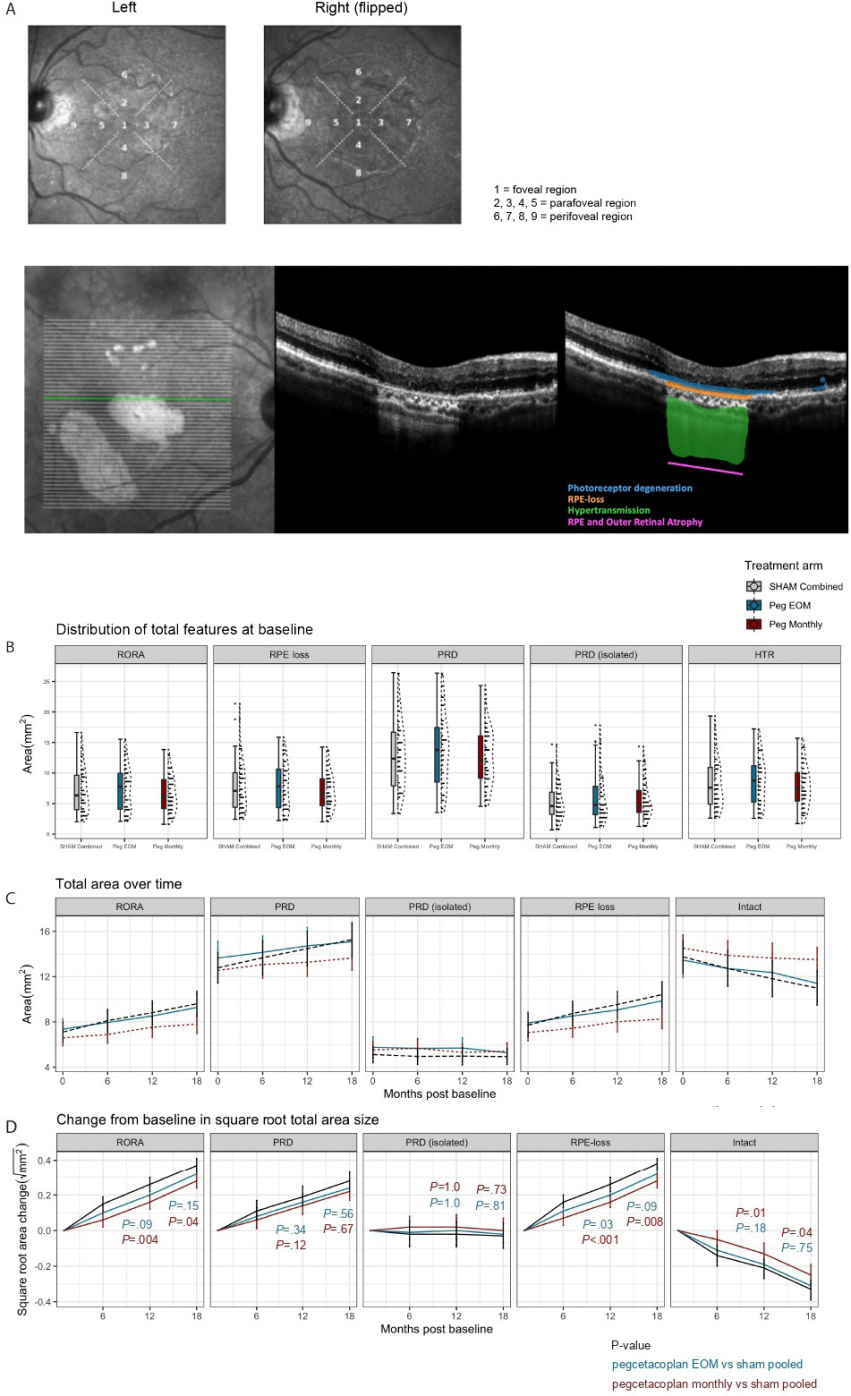
(A) Exemplar infrared en face image scan of left and right (flipped) fundus with Early Treatment of Diabetic Retinopathy Study (ETDRS) regions superimposed. The dimensions of the ETDRS circles are as follows: foveal (1 mm in diameter), perifoveal (3 mm in diameter) and parafoveal (6 mm in diameter). ‘Total area’ refers to the area of a given feature within the ETDRS circle. All B scans of a given optical coherence tomography were segmented automatically with the deep-learning models for photoreceptor degeneration (PRD; blue), RPE loss (orange) and hypertransmission (HTR; green). Overlapping regions of photoreceptor degeneration, RPE loss and hypertransmission as per A-scan were taken to be RPE loss and outer retinal atrophy (RORA; pink). PRD without any other overlapping features was labelled as PRD isolated (asterisk). (B) Distribution of total features at baseline (RORA, RPE loss, PRD, PRD isolated and HTR) by treatment group. There were no statistically significant differences in the baseline area across the three treatment arms (pegcetacoplan monthly, pegcetacoplan every other month and sham pooled groups). (C) Graph depicting the change from baseline in total area size (mm^2^) of RORA, PRD, PRD (isolated), RPE loss and intact macula in the study eye by month post baseline. (D) Graph showing least squares means and their 95% CIs by treatment group and month post baseline, which were estimated from a mixed effect model that included treatment and visit as factors and baseline RORA lesion as a covariate, as well as the interaction term of treatment×visit and visit×baseline. ***p value<0.001, **p value<0.01, *p value <0.05. HTR, hypertransmission; PRD, photoreceptor degeneration; RORA, RPE and outer retinal atrophy; RPE, retinal pigment epithelium.

This quantitative OCT (qOCT)-based deep-learning approach segments and quantifies GA and its constituent features from all OCT volumes as previously described (figure 2a and online supplemental materials).^17^ Briefly, a probability for a given feature is assigned to each voxel within an OCT volume. Features included: RPE loss, PRD, hypertransmission and RORA (defined as overlapping regions of RPE loss, PRD and hypertransmission, ie, any retinal areas with all three features present and therefore encompassing both incomplete and complete RORA).^10 21^ Combined representation of each feature both axially and topographically enabled consideration of: PRD in isolation (defined as PRD without overlapping RPE loss or hypertransmission); and intact macula (macular areas free of GA features). Of note, to enable longitudinal comparison of OCT time series from each study participant over 18 months of follow-up, we performed meticulous registration of OCT volumes across time points. A deep-learning model for foveal localisation developed by our research team was also applied to guide the accurate positioning of the 6 mm Early Treatment of Diabetic Retinopathy Study (ETDRS) grid. Quality control by manual inspection of foveal localisation and ETDRS grid positioning was undertaken by expert Reading Centre graders. All feature-specific surface area measurements were conducted within the ETDRS surface area and its subfields. The term ‘intact macula’ refers to the portion of the macula unaffected by any of the CAM-defined constituent features of GA and contained within the 6 mm diameter ETDRS grid centred on the fovea.

10.1136/bjo-2022-322672.supp4 Supplementary data

### Study outcomes

The primary endpoint was the least square (LS) mean change from baseline in square root transformed area (mm) of RORA in each of the three treatment arms (PM, PEOM and sham pooled) at 12 months.^22^ RORA is used to enable analyses requiring a continuous variable while encompassing both early atrophic changes (iRORA) and GA (cRORA).^17^ Secondary endpoints included square root change in area of CAM-defined constituent features of RORA—RPE loss, hypertransmission and PRD. Isolated PRD and intact macula (within the ETDRS grid) were introduced as novel clinical outcomes. In addition to area (mm^2^) by feature, baseline measurements included demographic information, perimeter of the lesion and its focality. Predictive values for GA growth of the total PRD to RPE loss ratio; of PRD in isolation; and of intact macula (all measured at baseline) were also study endpoints.

Due to GA’s non-uniform topographical distribution and its propensity for extra-foveal sites,^23^ feature-specific area analysis was conducted by ETDRS region of interest (foveal, perifoveal and parafoveal as well as regions 1–9) to evaluate change in area of these features over time in the form of square root change in area and per cent occupancy (measured as area of degeneration/total area of individual region).

### Statistical analyses

All data analyses were conducted using R (https://www.r-project.org/) provided in the public domain by R Core team 2020 R Foundation for Statistical Computing, Vienna, Austria.^24^ Most features at baseline were not normally distributed (inspected visually with quantile–quantile plots and Shapiro-Wilk normality test p value<0.05); therefore, the Wilcoxon rank test was used for baseline comparisons. Statistical tests were two-sided with alpha=0.05. LS means and their 95% CIs were estimated from a mixed effect model with a random intercept at the level of the patient that included treatment and visit as factors and baseline RORA lesion as a covariate, as well as cross-level interactions of treatment×visit and visit×baseline.

### Predicting GA growth

Per annum GA growth rate was calculated as the difference in RORA area from baseline to month 12 and considered as a dependent variable in a regression model. Coefficient of determination (R^2^) was computed and reported from 100-fold bootstrapped multivariable regression models.^25^ Independent variables are: ratio of total PRD to RPE loss at baseline; area of isolated PRD at baseline; and intact macula at baseline.

## Results

### Cohort and baseline characteristics

Among the 197 participants, the mean age was 79.5 years, baseline visual acuity 54.7 ETDRS letters and 62.9% were women (table 1). At baseline, RORA occupied a mean (SD) area of 7.00 mm^2^ (3.39) or 24.8% of the central macula as marked by the ETDRS region of 6 mm diameter. There was an average of 3.14 (2.32) distinct RORA lesions per study eye with an average total perimeter of 24.1 mm (11.1). Constituent features of GA and other novel anatomical features—including PRD, RPE loss, hypertransmission, PRD in isolation and intact macula—are described in table 1 and figure 2B.

**Table 1:** Demographic and baseline characteristics by treatment arm

Characteristic	Sham pooled (N=65)	PEOM (N=61)	P value versus sham	PM (N=71)	P value versus sham	All (N=197)
Gender						
Female	39 (60.0%)	40 (65.6%)		45 (63.4%)		124 (62.9%)
Male	26 (40.0%)	21 (34.4%)		26 (36.6%)		73 (37.1%)
Age (years)						
Mean (SD)	77.9 (7.58)	80.3 (7.43)		80.4 (7.44)		79.5 (7.53)
Median (Min, Max)	78.0 (60.0, 96.0)	80.0 (60.0, 97.0)		81.0 (63.0, 95.0)		80.0 (60.0, 97.0)
Baseline VA (ETDRS letters)						
Mean (SD)	52.4 (22.5)	56.3 (20.1)		55.5 (19.0)		54.7 (20.5)
Median (Min, Max)	58.0 (0, 85.0)	60.0 (4.00, 89.0)		58.0 (0, 83.0)		59.0 (0, 89.0)
RORA (mm^2^)						
Mean (SD)	7.10 (3.63)	7.36 (3.62)	0.660	6.59 (2.94)	0.578	7.00 (3.39)
Median (Min, Max)	6.33 (2.02, 16.6)	7.74 (2.08, 15.5)		6.05 (1.59, 13.8)		6.33 (1.59, 16.6)
PRD (mm^2^)						
Mean (SD)	12.8 (5.51)	13.6 (5.89)	0.557	12.5 (4.64)	0.794	13.0 (5.33)
Median (Min, Max)	12.3 (3.35, 26.4)	13.8 (3.53, 26.4)		12.0 (4.54, 24.3)		12.6 (3.35, 26.4)
PRD in isolation (mm^2^)						
Mean (SD)	5.12 (2.87)	5.76 (3.71)	0.680	5.53 (2.96)	0.680	5.47 (3.18)
Median (Min, Max)	4.59 (0.690, 14.7)	4.77 (1.08, 17.8)		4.50 (1.25, 14.3)		4.61 (0.690, 17.8)
RPE loss (mm^2^)						
Mean (SD)	7.71 (4.03)	7.91 (3.82)	0.756	7.06 (3.04)	0.440	7.54 (3.64)
Median (Min, Max)	7.09 (2.43, 21.4)	7.79 (2.24, 15.9)		6.54 (1.99, 14.3)		6.92 (1.99, 21.4)
Hypertransmission (mm^2^)						
Mean (SD)	8.18 (3.92)	8.67 (3.93)	0.601	7.84 (3.34)	0.601	8.21 (3.72)
Median (Min, Max)	7.60 (2.60, 19.3)	8.73 (2.57, 17.2)		7.39 (1.73, 15.7)		7.80 (1.73, 19.3)
Intact macula (mm^2^)						
Mean (SD)	13.7 (5.92)	13.5 (5.97)	0.777	14.5 (4.99)	0.649	13.9 (5.61)
Median (Min, Max)	13.9 (0.202, 24.0)	13.2 (0.360, 24.1)		14.1 (1.67, 23.6)		13.9 (0.202, 24.1)
Perimeter (mm)						
Mean (SD)	23.9 (10.3)	24.7 (11.2)	0.955	23.8 (11.7)	0.955	24.1 (11.1)
Median (Min, Max)	21.9 (7.19, 60.9)	24.4 (7.56, 58.4)		19.6 (7.21, 55.3)		21.9 (7.19, 60.9)
Focality						
Mean (SD)	2.94 (2.08)	3.43 (2.48)	0.571	3.07 (2.38)	0.741	3.14 (2.32)
Median (Min, Max)	2.00 (1.00, 10.0)	3.00 (1.00, 10.0)		2.00 (1.00, 12.0)		2.00 (1.00, 12.0)

ETDRS, Early Treatment of Diabetic Retinopathy Study; PEOM, pegcetacoplan every other month; PM, pegcetacoplan monthly; PRD, photoreceptor degeneration; RORA, RPE and outer retinal atrophy; RPE, retinal pigment epithelium; VA, Visual Acuity.

At baseline, no statistically significant differences were found between the treatment arms (either of the pegcetacoplan arms and pooled sham arm) for any demographic or GA features across the entire ETDRS area (table 1, and figure 2) across ETDRS regions 1–9 (online supplemental figure S1 and online supplemental table S1) or regions grouped by distance from the fovea (online supplemental table S2 and online supplemental figure S2). When taken collectively, the average baseline occupancy of RORA was greater in the foveal region (59.7%) than the parafoveal region (58.5%), which was greater than the perifoveal region (13.5%) (online supplemental table S2 and online supplemental figure S2).

10.1136/bjo-2022-322672.supp1 Supplementary data

10.1136/bjo-2022-322672.supp5 Supplementary data

10.1136/bjo-2022-322672.supp6 Supplementary data

10.1136/bjo-2022-322672.supp2 Supplementary data

### Longitudinal segmentations

In this analysis’ main endpoint (12 months post baseline), a statistically significant reduction in GA growth rate was observed between PM and pooled sham (0.151 and 0.277 mm, respectively; p=0.004) (figure 2C,D, table 2A and online supplemental figure S3A). A lower treatment effect was observed when comparing PEOM with sham arm, although not statistically significant (0.202 and 0.277 mm, p=0.09). This corresponds to a reduction of 45% in growth rate of cRORA between PM and sham and 27% reduction between PEOM and sham. At 18 months following baseline (6 months following treatment cessation), those previously on monthly pegcetacoplan maintained a statistically significant reduction in cRORA growth compared with sham pooled (0.251 and 0.396 mm, p=0.04).

10.1136/bjo-2022-322672.supp3 Supplementary data

**Table 2:** Change in square root transformed area from baseline for RORA and each feature at 6, 12 and 18 months

(A) Change in square root area of RORA from baseline to 18 months (mm)						
	Sham pooled	P value	PEOM	P value	PM	P value
6 months	N=61		N=61		N=63	
Mean (SD)	0.162 (0.152)	N/A	0.104 (0.170)	N/A	0.0510 (0.144)	N/A
Median (Min, Max)	0.137 (−0.268, 0.691)		0.098 (−0.642, 0.533)		0.0496 (−0.479, 0.389)	
12 months	N=58		N=52		N=56	
Mean (SD)	0.277 (0.209)	N/A	0.202 (0.256)	0.09	0.151 (0.142)	0.004
Median (Min, Max)	0.23 (−0.244, 0.810)		0.168 (−0.422, 0.921)		0.14 (−0.250, 0.587)	
18 months	N=58		N=55		N=62	
Mean (SD)	0.396 (0.256)	N/A	0.315 (0.395)	0.15	0.251 (0.193)	0.04
Median (Min, Max)	0.347 (−0.215, 1.04)		0.293 (−1.25, 1.30)		0.242 (−0.193, 0.937)	

(B) Change in square root area of PRD from baseline to 18 months (mm)						
	Sham pooled	P value	PEOM	P value	PM	P value
6 months	N=61		N=61		N=63	
Mean (SD)	−0.0363 (0.255)	N/A	−0.00442 (0.362)	N/A	0.0243 (0.387)	N/A
Median (Min, Max)	−0.0358 (−1.02, 0.575)		−0.0104 (−0.871, 2.15)		−0.0203 (−0.659, 1.77)	
12 months	N=58		N=52	0.34	N=56	0.12
Mean (SD)	−0.0185 (0.316)	N/A	−0.00234 (0.404)		0.000414 (0.431)	
Median (Min, Max)	0.0124 (−1.33, 0.457)		−0.0395 (−0.962, 1.57)		−0.0413 (−1.26, 2.22)	
18 months	N=58		N=55	0.56	N=62	0.67
Mean (SD)	−0.0296 (0.375)	N/A	−0.0533 (0.426)		0.0417 (0.486)	
Median (Min, Max)	−0.0512 (−0.824, 1.27)		−0.085 (−1.05, 1.48)		0.00812 (−1.04, 2.27)	

(C) Change in square root area of PRD in isolation from baseline to 18 months (mm)						
	Sham pooled	P value	PEOM	P value	PM	P value
6 months	N=61		N=61		N=63	
Mean (SD)	0.0975 (0.191)	N/A	0.0790 (0.273)	N/A	0.0740 (0.322)	N/A
Median (Min, Max)	0.13 (−0.580, 0.496)		0.0757 (−0.814, 1.45)		0.0664 (−0.520, 1.60)	
12 months	N=58		N=52		N=56	
Mean (SD)	0.219 (0.238)	N/A	0.155 (0.241)	1	0.113 (0.333)	1
Median (Min, Max)	0.207 (−0.657, 0.730)		0.107 (−0.492, 0.896)		0.0837 (−1.01, 1.81)	
18 months	N=58		N=55		N=62	
Mean (SD)	0.301 (0.278)	N/A	0.217 (0.400)	0.81	0.224 (0.352)	0.73
Median (Min, Max)	0.299 (−0.496, 1.08)		0.212 (−1.44, 0.993)		0.183 (−0.778, 1.83)	

(D) Change in square root area of RPE loss from baseline to 18 months (mm)						
	Sham pooled	P value	PEOM	P value	PM	P value
6 months	N=61		N=61		N=63	
Mean (SD)	0.161 (0.151)	N/A	0.107 (0.155)	N/A	0.0761 (0.153)	N/A
Median (Min, Max)	0.134 (−0.258, 0.645)		0.0973 (−0.446, 0.579)		0.0565 (−0.293, 0.493)	
12 months	N=58		N=52		N=56	
Mean (SD)	0.287 (0.197)	N/A	0.198 (0.242)	0.03	0.147 (0.158)	<0.001
Median (Min, Max)	0.241 (−0.235, 0.830)		0.168 (−0.368, 1.06)		0.15 (−0.276, 0.587)	
18 months	N=58		N=55		N=62	
Mean (SD)	0.410 (0.264)	N/A	0.314 (0.369)	0.09	0.242 (0.200)	0.008
Median (Min, Max)	0.384 (−0.202, 1.13)		0.291 (−0.997, 1.25)		0.247 (−0.202, 0.904)	

(E) Change in square root area of hypertransmission from baseline to 18 months (mm)						
	Sham pooled	P value	PEOM	P value	PM	P value
6 months	N=61		N=61		N=63	
Mean (SD)	0.139 (0.153)	N/A	0.130 (0.206)	N/A	0.0816 (0.157)	N/A
Median (Min, Max)	0.12 (−0.339, 0.635)		0.113 (−0.744, 0.881)		0.0917 (−0.414, 0.508)	
12 months	N=58		N=52		N=56	
Mean (SD)	0.239 (0.192)	N/A	0.220 (0.233)	0.82	0.211 (0.212)	0.82
Median (Min, Max)	0.214 (−0.329, 0.747)		0.188 (−0.167, 0.960)		0.184 (−0.231, 1.01)	
18 months	N=58		N=55		N=62	
Mean (SD)	0.356 (0.223)	N/A	0.306 (0.366)	0.38	0.288 (0.190)	0.38
Median (Min, Max)	0.349 (−0.301, 1.00)		0.289 (−1.49, 0.999)		0.27 (−0.189, 0.750)	

(F) Change in square root of intact macula area from baseline to 18 months (mm)						
	Sham pooled	P value	PEOM	P value	PM	P value
6 months	N=61		N=61		N=63	
Mean (SD)	−0.124 (0.238)	N/A	−0.111 (0.328)	N/A	−0.0733 (0.303)	N/A
Median (Min, Max)	−0.123 (−0.867, 0.570)		−0.0793 (−1.74, 0.870)		−0.0447 (−1.44, 0.550)	
12 months	N=58		N=52		N=56	
Mean (SD)	−0.259 (0.291)	N/A	−0.180 (0.306)	0.18	−0.0878 (0.319)	0.01
Median (Min, Max)	−0.249 (−0.893, 0.558)		−0.136 (−0.991, 0.805)		−0.0807 (−1.64, 1.02)	
18 months	N=58		N=55		N=62	
Mean (SD)	−0.354 (0.316)	N/A	−0.339 (0.384)	0.75	−0.207 (0.312)	0.04
Median (Min, Max)	−0.358 (−1.24, 0.510)		−0.302 (−1.34, 0.775)		−0.179 (−1.64, 0.803)	

Table shows mean square root change of area, SD and p values for comparison of pegetacoplan EOM or monthly versus sham pooled when considering (A) RORA, (B) PRD, (C) PRD in isolation from baseline to 18 months, (D) RPE loss from baseline to 18 months, (E) hypertransmission and (F) intact macula.

EOM, every other month; N, number of study eyes; PM, pegcetacoplan monthly; PRD, photoreceptor degeneration; RORA, RPE and outer retinal atrophy; RPE, retinal pigment epithelium.

**Figure 3:**
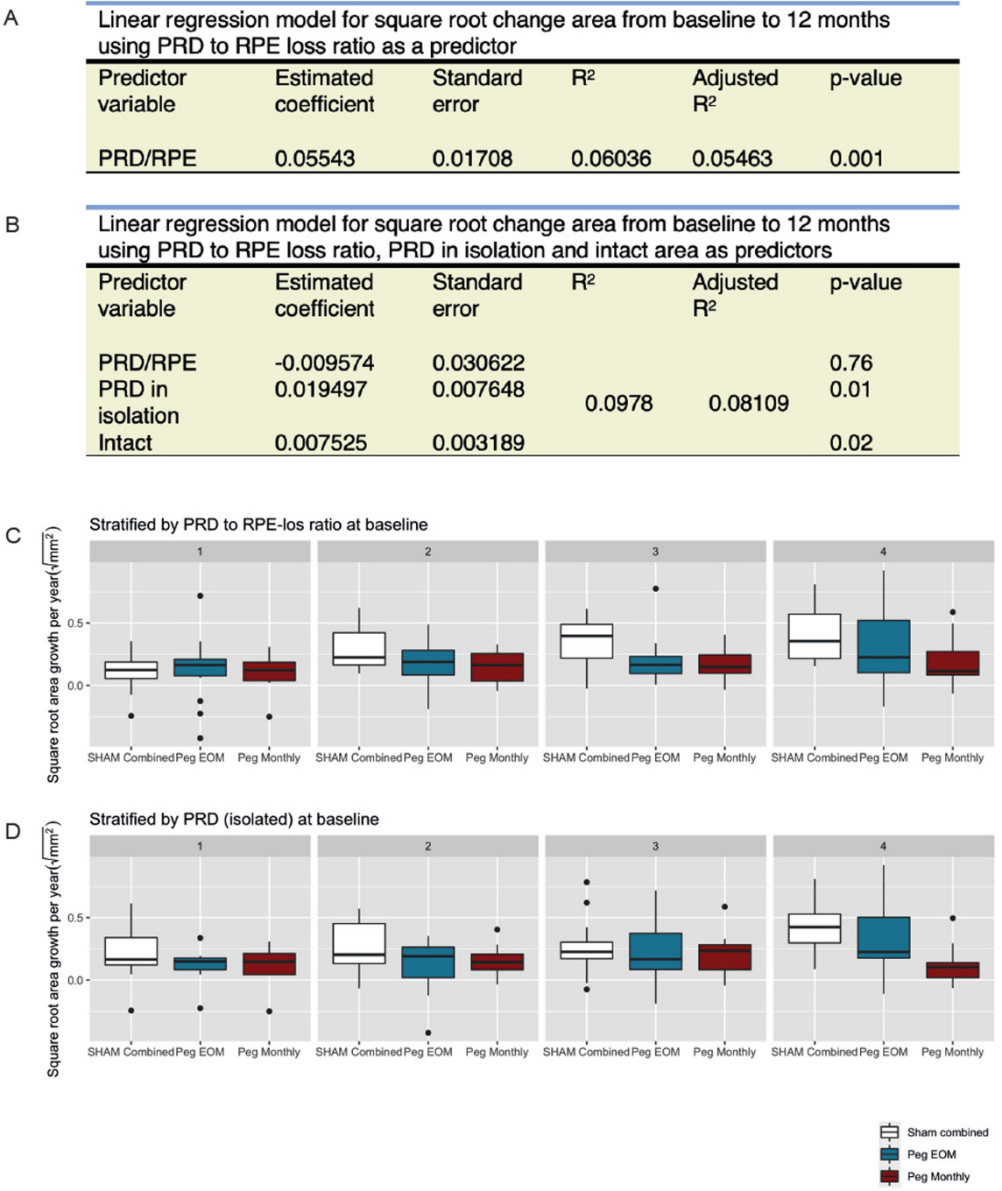
Predictors of RORA progression. (A) Linear regression model for square root change area from baseline to 12 months using PRD to RPE loss ratio as a predictor. (B) Linear regression model for square root change area from baseline to 12 months using PRD to RPE loss ratio, PRD in isolation and intact macula as predictors. (C) Boxplot of square root area growth per year (mm) in each treatment arm stratified by PRD to RPE loss ratio at baseline. (D) Boxplot of square root area growth per year (mm) in each treatment arm stratified by PRD (isolated) at baseline. EOM, every other month; Peg, pegcetacoplan; PRD, photoreceptor degeneration; RORA, RPE and outer retinal atrophy; RPE, retinal pigment epithelium.

A similar pattern emerged when considering individual CAM-defined constituent features of GA (figure 2C,D). At 12 months, RPE loss demonstrated a slower growth rate compared with sham pooled in both PM (0.147 and 0.287 mm, p=<0.001) and PEOM (0.198 and 0.287 mm, p=0.03). This was similarly observed at 18 months for PM versus sham pooled (0.410 and 0.242 mm, p=0.008) (table 2D). The area of intact macula (within the ETDRS grid) showed an inverse trend, with significantly slower loss of intact macula in PM versus sham pooled at 12 and 18 months (−0.0878 and −0.259 mm, p=0.01; and −0.207 and −0.354 mm, p=0.04, respectively) (table 2E). While a similar trend was observed when comparing the PEOM group with sham pooled, a statistically significant difference was not found for intact macula. The mean growth rate of PRD also generally followed a dose-dependent effect of pegcetacoplan; however, a statistically significant change was not observed.

The analysis was repeated for each of the ETDRS regions (online supplemental table S2) and summed according to their distance from the fovea. Parafoveal and perifoveal regions correlated with the overall trends, though the effect was less clear in the foveal region (online supplemental figure S3B).

### Predicting GA growth

The ratio of PRD to RPE loss has been demonstrated to predict RPE loss in GA.^25^ Here, we expand on the previous finding, showing that PRD/RPE loss at baseline also correlates with GA growth over 12 months (R^2^ 0.0554, p=0.001). We next asked whether the predictive effect of baseline PRD/RPE loss ratio can be accounted for by PRD in isolation. This was interrogated using a multivariable linear regression that included baseline PRD/RPE loss ratio, area of isolated PRD and intact macula at baseline as independent variables. PRD in isolation and intact macula emerged as statistically relevant predictors of GA growth at 12 months (coefficient 0.0195, p=0.01 and 0.00752, p=0.02, respectively; figure 3). Their predictive value is further supported by considering GA growth at 12 months by stratifying baseline PRD/RPE loss ratio and isolated PRD into quartiles (figure 3C). The greatest growth was seen at the highest quartiles for each variable. Interestingly, we also observed the highest treatment effect in the highest quartiles for both baseline PRD/RPE loss ratio and isolated PRD.

## Discussion

### Key findings

This post hoc analysis uses a novel validated deep-learning platform to demonstrate the effect of pegcetacoplan on key features of GA as imaged with SD-OCT. Our analyses support the finding that local complement inhibition with pegcetacoplan reduces the growth of GA compared with sham treatment.^26^ Compared with sham pooled arms, monthly pegcetacoplan significantly reduced GA growth and preserved intact macula (within ETDRS grid) over the 12-month observation period; this effect was maintained for at least 6 months following treatment cessation. Here, PRD in isolation and intact macula was identified as independent statistically relevant predictors of future GA growth. Of note, the growth rate reducing effect of pegcetacoplan was most pronounced in the parafoveal region of the ETDRS grid. The effect was less pronounced in the perifoveal region or the central foveal region (potentially due to high foveal occupancy by GA at baseline), combined with the relatively small sample, which is not conducive to revealing subtle effects.

### Interrogating pegcetacoplan efficacy with FAF and SD-OCT

The primary end point of the FILLY trial was the change from baseline in square root GA lesion size as measured with FAF.^22^ Despite the recognised advantages of OCT over FAF, the feasibility of SD-OCT imaging to inform clinical trial endpoints in GA has been uncertain due to the labor-intensive nature and time constraints for B-scan-level manual image segmentation, interobserver variability and specialist training required for expert Reading Centre graders.^10^ Our qOCT platform overcomes these practical limitations using a deep-learning model that automatically segments each CAM-defined feature of GA and renders them to reveal a spectrum of GA stages.

We confirmed that, given a robust analytical method, qOCT findings support the primary FAF endpoints of the FILLY trial. The FILLY trial demonstrated a mean change of 0.25, 0.28 and 0.36 mm for PM, EOM and sham treatment (p=0.01 and p=0.06, respectively). Similarly, with qOCT we observed this for GA (0.151, 0.202 and 0.277 mm for monthly, EOM and sham, respectively) but also for RPE loss and intact macula, yet not for hypertransmission, PRD and PRD in isolation. Parallels in growth rate can be drawn between RPE loss and FAF signal, suggesting the FAF signal may primarily reflect the effect of RPE loss itself. The qOCT approach therefore expands and provides nuance to our understanding of GA growth beyond just RPE loss.

### Proposed mechanism of pegcetacoplan and GA pathophysiology

Evidence suggests that GA begins with degeneration of photoreceptors followed by RPE loss.^20^ This is supported by our findings that PRD in isolation exists and—along with intact macula—is a key predictor of future RORA (starting with iRORA and progressing to cRORA/GA). Furthermore, pegcetacoplan treatment is associated with relative preservation of intact macula over time, indicating that treatment may halt the process towards the initial photoreceptor insult. Additionally, slowing of the first detectable degenerative step from PRD to RPE loss suggests that pegcetacoplan can be protective against further damage even after the macula has undergone early stages of degeneration. Monthly intravitreal therapy conferred the most pronounced protective effect.

These combined data suggest that complement inhibition with pegcetacoplan acts sufficiently upstream in GA pathogenesis to protect intact macula from initial degeneration of photoreceptors. There is histopathological and genetic evidence supporting this hypothesis. Modulation of complement cascade activity in general, and specifically downregulation of C3, has been linked to increased photoreceptor survival and improved visual function in a C3 knockout mouse model.^27^ Immunohistochemistry of both primate and human retinas has shown C3 deposition in the choriocapillaris and, perhaps more surprisingly given the blood–retina barrier, also in the outer photoreceptor segments.^28–31^ The protective effect of C3 inhibition could therefore be indirect at the choriocapillaris level, or direct at the level of the photoreceptors.^32^ This is underpinned by in vitro evidence of C3b-regulated macrophage phagocytosis.^33^

Our deep-learning methodology made it possible to quantify PRD in isolation and assess its potential as a predictor of disease progression. It has been suggested that the PRD/RPE loss ratio is predictive of future RPE loss,^25^ and our data supported this and expanded it further to apply also to GA growth. Importantly, the area of isolated PRD remained relatively constant in relation to the size of established RORA. The effect observed may thus be largely dependent on the amount of PRD in isolation and the amount of intact macula ‘available’ in absolute terms, making PRD in isolation a good predictor of future RORA (figure 4B). This analysis provides a more profound interpretation for the role of PRD/RPE loss ratio, indicating that the determining predictive variable is in fact the area of PDR without underlying RPE loss (PDR in isolation) as well as the area unaffected by either PRD or RPE loss at baseline.

### Efficacy by macular topography

A key asset of the deep-learning model is the ability to topographically chart the progression of GA. The macula is not a uniform structure, and the development of GA is non-homogeneous. Observational studies have suggested the initial steps of GA take place outside of the fovea, specifically in the perifoveal region of the macula,^23 34^, which offers the possibility of non-uniform pegcetacoplan action. Automatic registration and annotation allowed us to compare both within and between study subjects, thus showing the effect of pegcetacoplan in the foveal, parafoveal and perifoveal areas.

We did not find a statistically significant protective effect of pegcetacoplan on the foveal region. This is likely due to the high foveal occupancy, that is, 59.7% of the foveal region was affected by GA at baseline and therefore there was little healthy macula remaining to allow detecting protective treatment effects. In contrast to our findings, Vogl *et al* ^35^ indicate a greater effect of pegcetacoplan in reducing the expansion rate of GA towards the foveal region as opposed to the peripheral GA lesion boundary. The proposed methodology by Vogl *et al* is intriguing, yet highly convoluted and complex, combining several previous methodologies. Most previous methodologies were developed for entirely different use cases than the one considered in this work. For example, the local progression rate was designed for an OCT-angiography use case. Similarly, the automated quantification of photoreceptor alteration ensemble model was previously developed by the same group, trained on just 26 OCT volumes (all of which originating from cases of diabetic retinopathy and retinal vein occlusions, none from cases of GA). Moreover, the segmentations themselves on the 26 OCT volumes, which were then used to train the ensemble deep-learning model, were semiautomated segmentations generated by an older generation ‘graph-theoric’ method (non-AI), which itself relied on 13 OCT volumes for its development. Additionally, the areas of GA were determined using a deep-learning model assessing exclusively the RPE and none of the other features recommended by the CAM-consensus definitions for the identification of GA. Finally, the output of these (and others) unrelated methodological approaches were combined using Generalised Additive Mixed-Effect Models, which, at the admission of the authors, are hugely challenging in their interpretation requiring numerous interjected assumptions by the authors themselves about their actual meaning. Although an intriguing exploratory approach, we interpret the conclusions of this report with great caution. We have employed exclusively established, reliable methodologies in our current work, which do not permit assessing with confidence the effect of pegcetacoplan in the central macular direction. We suggest interrogating the effect of pegcetacoplan on a group of patients with less advanced foveal disease. Going forward, this methodology permits tracking of GA areas over time for personalised GA monitoring, as well as lesion registration for interpatient modelling and research.

## Limitations

Even though GA is a disease of the macula, there are extramacular regions not captured by the OCT that cannot be segmented automatically. In instances of extramacular pathology or other pathology complicating GA, a different form of analysis should be employed. The study was also conducted as per the trial protocol (only including patients with relevant OCT imaging), which made detecting smaller effects unlikely due to lack of power. The high proportion of the foveal region already affected by GA at baseline did not allow drawing conclusions on the potential rate limiting effect of pegcetacoplan on GA expansion towards the fovea. The application of our automated segmentation model was limited to Heidelberg OCTs, which constitute the great majority of the FILLY study dataset. The expansion of model application to Zeiss and TopCon OCTs is in active process of development by our research team.

## Conclusion

Pegcetacoplan is a novel therapeutic with accruing evidence of efficacy, and this post hoc analysis presents results at the SD-OCT level that further confirm its treatment effect. In assessing the efficacy of several emerging therapeutics for GA, the common rate-limiting step is efficiently extracting structural data from imaging, and here, we have provided a solution with a fully automated deep-learning approach. Incorporating topographical information provided greater insight into the effect of treatment beyond binary presence or absence of features, and volumetric analysis made it feasible to instantly classify each case along the spectrum of GA subtypes according to the most widely established international CAM consensus definitions. Finally, we identified two novel clinical endpoints, PRD in isolation and intact macula, that are predictors of GA progression. They can thus contribute to more efficient assessment of therapeutics for early atrophic changes and merit consideration as clinical endpoints in future studies of early-stage GA.
