# Supplementary material for: Deep-learning automated quantification of longitudinal OCT scans demonstrates reduced RPE loss rate, preservation of intact macular area and predictive value of isolated photoreceptor degeneration in geographic atrophy patients receiving C3 inhibition treatment
Source: Br J Ophthalmol. 2023 Apr 24;108(4):536–45. doi: 10.1136/bjo-2022-322672 (PMC10958254; doi:10.1136/bjo-2022-322672)
Supplement: Supplementary data [file bjo-2022-322672supp004.pdf]

# Supplementary materials

**Supplemental Methods: Image pre- and post-processing**

Each B-scan was segmented 50 times (once in its un-augmented format and 49 times following basic augmentations as previously described[17]) with the averaged segmentation taken forward. Voxel spatial localisation was interpolated in relation to the central fovea, thereby standardising topographical location and enabling inter-eye comparison, as well as intra-eye comparison between timepoints (**Figure 2a**). Central foveal points were automatically annotated (*manuscript in preparation*) with subsequent quality assurance review by a reading centre expert grader at the Moorfields Reading Centre with manual correction when required. The spatial localisation of feature probabilities was also considered by dividing the macula into each of the Early Treatment Diabetic Retinopathy Study (ETDRS) regions, i.e. mean probability within each of the nine ETDRS regions (foveal, 4 parafoveal, and 4 perifoveal areas for the nasal, temporal, superior, and inferior regions). The area of each segmented feature in square millimetres ($mm^2$) was considered by applying optimized probability thresholds identified from the original model development and validation.[17]

to be established, the consistent findings across multiple studies once again reinforce this key anatomic characteristic as a predictor of greater GA progression.

**Supplemental Methods: Deep-Learning methodology for Automated OCT segmentation**

PyTorch was used to implement the network. The training was trained on a single Quadro P6000 GPU. The model used is an end-to-end fully convolutional network called U-net, which has a symmetric network architecture and contains both encoder and decoder. The encoder consists of a sequence 3 × 3 convolution layer and a 2 × 2 max-pooling layer with a stride of 2. The number of filters in the convolutional layers is doubled through repeating the above process and down-sampling. At the last layer, the encoder and decoder are connected by two 3 × 3 convolution operations. The decoder reduces the feature channels through 2 × 2 convolution layers followed by two 3 × 3 up-convolution layers. Halving the number of filters at each stage, the up-sampling operations and two convolution operations are repeated four times. Final segmentation map is generated by a 1 × 1 convolution operation with Sigmoid activation function. In this architecture, except for the final convolution operation, all the other convolution layers use the rectified linear unit activation function. Spatial information lost by pooling operations is retrieved by skip connections in the architecture. Prior to each pooling operation, the output of the encoder convolutional layer is transferred to the decoder. The output of the up-sampling operations is then concatenated with the transferred feature maps, which are then propagated to the successive layers.

OCT volumes were rescaled to the most common size in the development data - a width of 512 pixels and 5.8 mm (approximately 11 micrometer per pixel). Batches of 8 B-scans and corresponding annotations were fed to the model. Binary cross entropy loss and soft Dice loss were used as a loss-function with 0.5 weight for each to measure the dissimilarity between the current prediction of the model and the reference. The internal parameters of the model were then optimised using RMSprop.

Training was considered finished when performance on the loss of validation set did not improve for 2 epochs, where every epoch consisted of 378 updates. This happened after 14k, 14,4k, 11.7k, 12.1k updates for RPE-loss, photoreceptor degeneration, HTR, and GA respectively. The U-Net architecture consists of an encoder and a symmetric decoder. The encoder aggregates information from a large spatial context and converts it into an abstract representation. The decoder then generates the segmentation likelihood images based on this representation.

**Supplemental Results: Significant trends and their Interpretation**

This post hoc analysis did not find a statistically significant effect of pegcetacoplan on PRD or PRD in isolation, but there was a nominal decrease in average PRD in isolation between EOM and sham, and a greater one seen between monthly and sham treatment. This trend was present at 6, 12 and 18 months. The study estimated 240 participants would be required to have a 90% power to detect a 30% effect on overall lesion size. The disproportionate magnitude of RORA vs PRD changes would therefore likely result in a different power calculation. Interestingly, the rate of ‘loss of intact macula’ was statistically different between monthly pegcetacoplan and sham treatment. When the effect of pegcetacoplan was stratified by isolated PRD at baseline, higher baseline isolated PRD was associated with a more pronounced treatment effect. Based on these findings, pegcetacoplan may act to slow progression of PRD in GA.

From Steinle et al (2021), similar to our observations, faster GA progression in the SEATTLE trial was observed in eyes without subfoveal retinal pigment epithelial atrophy. In addition, the Geographic Atrophy Progression (GAP) study reported that GA growth over a year was significantly greater in patients with extrafoveal lesions compared with foveal lesions. The AREDS2 study reported faster GA enlargement of noncentral lesions in patients followed for more than 4 years. GA progression was also greater in patients with non subfoveal lesions in the Proxima studies over 2 years. Although the biological or mechanistic basis for faster progression of extrafoveal lesions is yet
