# Supplementary figures and images for: Deep-learning automated quantification of longitudinal OCT scans demonstrates reduced RPE loss rate, preservation of intact macular area and predictive value of isolated photoreceptor degeneration in geographic atrophy patients receiving C3 inhibition treatment
Source: Br J Ophthalmol. 2023 Apr 24;108(4):536–45. doi: 10.1136/bjo-2022-322672 (PMC10958254; doi:10.1136/bjo-2022-322672)

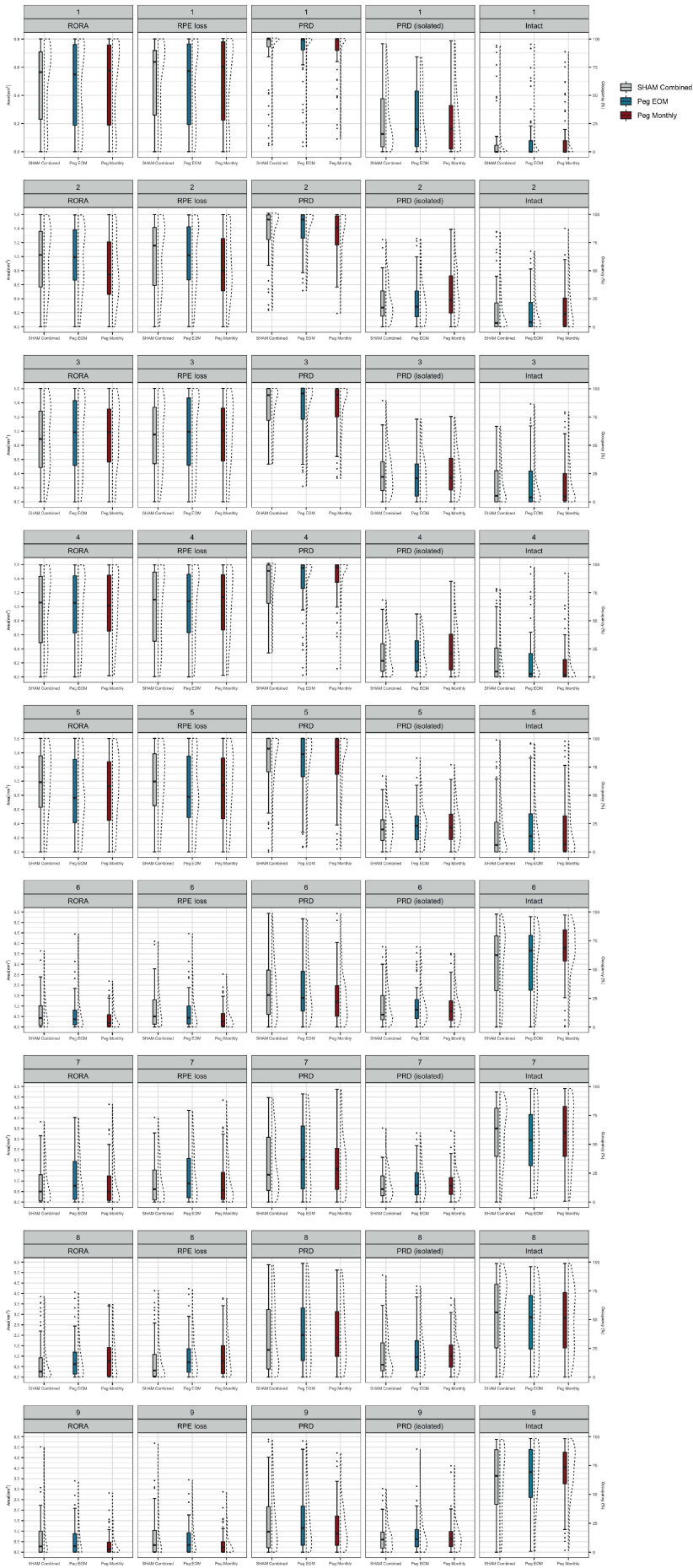

Supplement: Supplementary data [file bjo-2022-322672supp001.pdf]

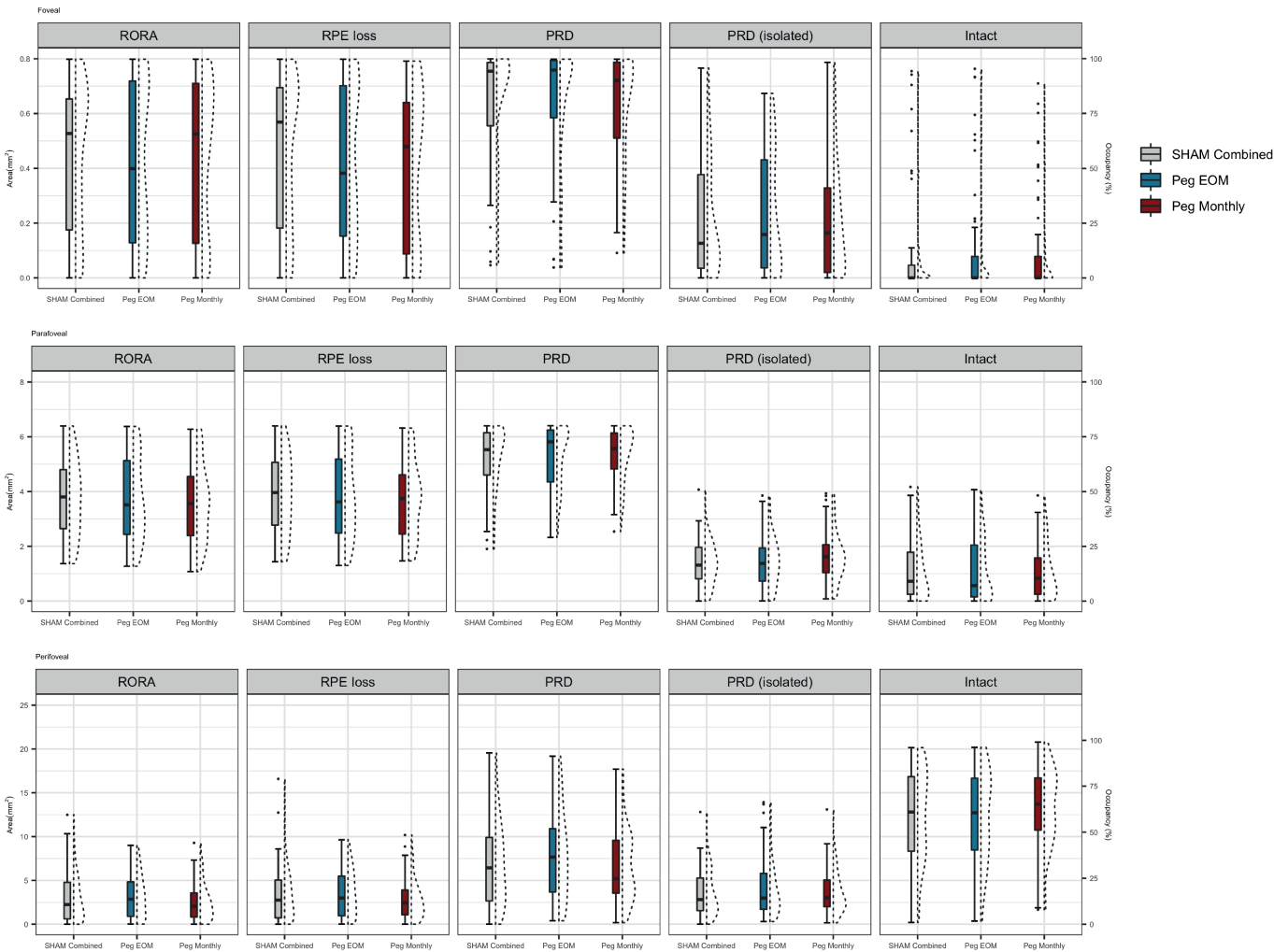

Supplement: Supplementary data [file bjo-2022-322672supp002.pdf]

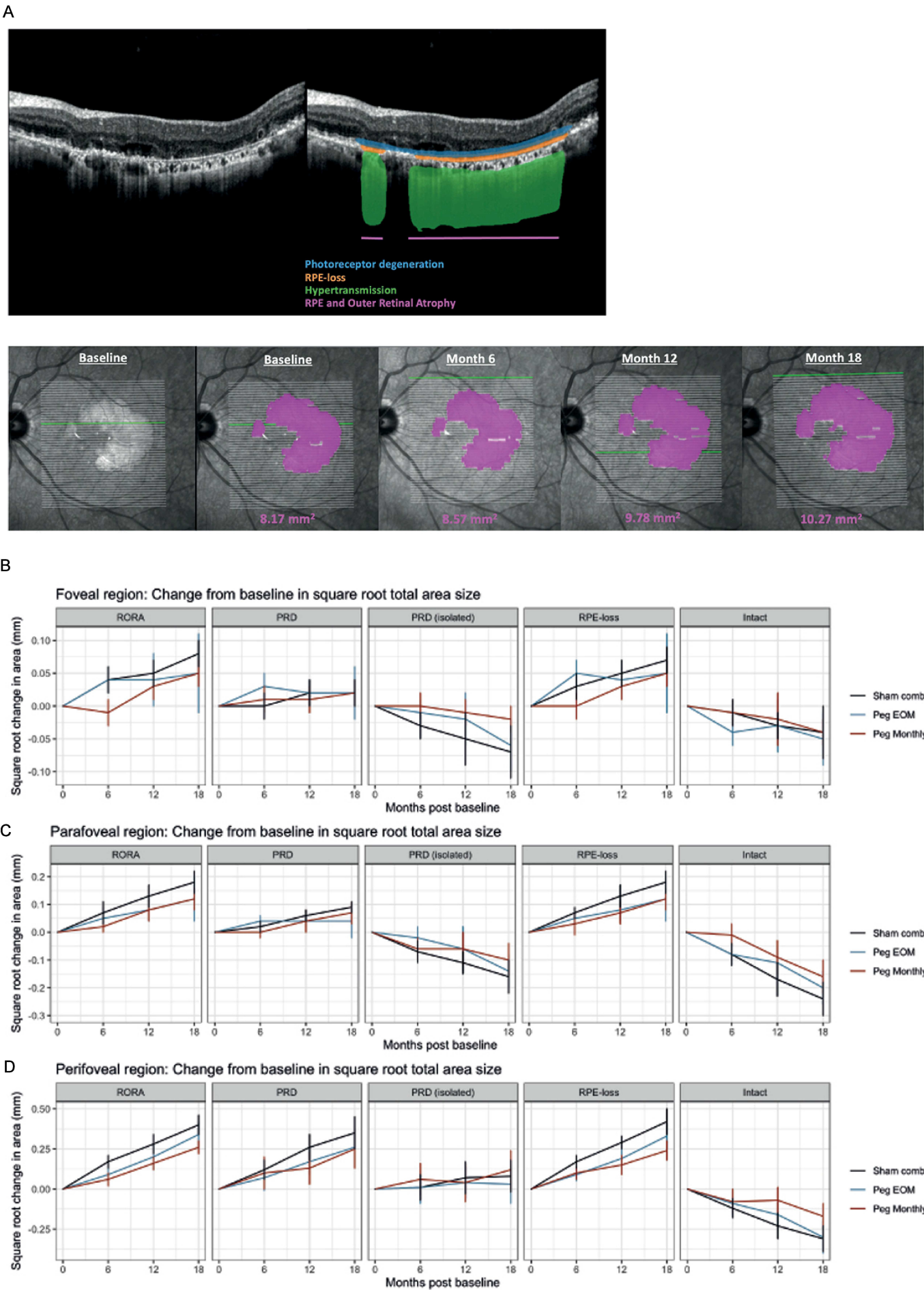

Supplement: Supplementary data [file bjo-2022-322672supp003.pdf]
