# Supplementary material for: Deep-learning automated quantification of longitudinal OCT scans demonstrates reduced RPE loss rate, preservation of intact macular area and predictive value of isolated photoreceptor degeneration in geographic atrophy patients receiving C3 inhibition treatment
Source: Br J Ophthalmol. 2023 Apr 24;108(4):536–45. doi: 10.1136/bjo-2022-322672 (PMC10958254; doi:10.1136/bjo-2022-322672)
Supplement: Supplementary data [file bjo-2022-322672supp005.pdf]

Supplementary Table 1. Baseline features of qOCT by treatment arm and ETDRS region.

**ETDRS region**		**1**			**2**			**3**	
**Treatment**	Sham Combined (N=65)	Pegcetacoplan EOM (N=61)	Pegcetacoplan Monthly (N=71)	Sham Combined (N=65)	Pegcetacoplan EOM (N=61)	Pegcetacoplan Monthly (N=71)	Sham Combined (N=65)	Pegcetacoplan EOM (N=61)	Pegcetacoplan Monthly (N=71)
**Characteristic**									
**RORA**									
Mean (SD)	0.470 (0.283)	0.455 (0.298)	0.487 (0.293)	0.934 (0.481)	0.964 (0.462)	0.817 (0.478)	0.889 (0.475)	0.942 (0.528)	0.910 (0.485)
Median [Min, Max]	0.564 [0, 0.801]	0.549 [0, 0.801]	0.577 [0, 0.801]	1.02 [0, 1.60]	0.993 [0, 1.59]	0.742 [0, 1.60]	0.889 [0.00140, 1.60]	0.986 [0.221, 1.61]	0.986 [0, 1.60]
**PRD**									
Mean (SD)	0.708 (0.196)	0.700 (0.202)	0.709 (0.174)	1.33 (0.379)	1.37 (0.300)	1.32 (0.319)	1.34 (0.325)	1.34 (0.378)	1.35 (0.331)
Median [Min, Max]	0.798 [0.0462, 0.807]	0.795 [0.0378, 0.804]	0.798 [0.0910, 0.804]	1.53 [0.237, 1.62]	1.53 [0.517, 1.60]	1.41 [0.192, 1.60]	1.51 [0.534, 1.60]	1.53 [0.221, 1.61]	1.52 [0.333, 1.60]
**PRD in isolation**									
Mean (SD)	0.214 (0.218)	0.228 (0.221)	0.204 (0.207)	0.348 (280)	0.360 (0.315)	0.453 (0.342)	0.406 (0.305)	0.363 (0.311)	0.412 (0.302)
Median [Min, Max]	0.126 [0, 0.766]	0.158 [0, 0.674]	0.164 [0, 0.787]	0.274 [0, 1.24]	0.287 [0, 1.26]	0.382 [0, 1.39]	0.353 [0, 1.43]	0.332 [0, 1.17]	0.342 [0, 1.21]
**RPE loss**									
Mean (SD)	0.496 (0.286)	0.472 (0.298)	0.506 (0.295)	0.987 (0.489)	1.01 (0.476)	0.865 (0.480)	0.931 (0.482)	0.977 (0.536)	0.940 (0.488)
Median [Min, Max]	0.637 [0, 0.801]	0.573 [0, 0.801]	0.602 [0, 0.804]	1.16 [0, 1.60]	1.02 [0, 1.59]	0.804 [0, 1.60]	0.952 [0.00140, 1.60]	0.992 [0, 1.60]	1.01 [0, 1.60]
**HTR**									
Mean (SD)	0.556 (0.252)	0.526 (0.271)	0.558 (0.276)	1.05 (0.466)	1.08 (0.393)	0.968 (0.426)	1.04 (0.436)	1.04 (0.481)	1.03 (0.470)
Median [Min, Max]	0.664 [0, 0.801]	0.604 [0, 0.804]	0.692 [0.00840, 0.801]	1.21 [0, 1.60]	1.07 [0.224, 1.59]	0.971 [0, 1.60]	1.15 [0.0168, 1.60]	1.12 [0, 1.60]	1.12 [0, 1.60]
**Intact macula**									
Mean (SD)	0.0845 (0.190)	0.101 (0.202)	0.0909 (0.174)	0.243 (0.366)	0.228 (0.301)	0.274 (0.317)	0.251 (0.314)	0.264 (378)	0.252 (0.331)
Median [Min, Max]	0 [0, 0.755]	0 [0, 0.763]	0 [0, 0.710]	0.0546 [0, 1.36]	0.0630 [0, 1.08]	0.182 [0, 1.40]	0.0854 [0, 1.07]	0.0644 [0, 1.38]	0.0784 [0, 1.27]

ETDRS region		4			5			6	
Treatment	Sham Combined (N=65)	Pegcetacoplan EOM (N=61)	Pegcetacoplan Monthly (N=71)	Sham Combined (N=65)	Pegcetacoplan EOM (N=61)	Pegcetacoplan Monthly (N=71)	Sham Combined (N=65)	Pegcetacoplan EOM (N=61)	Pegcetacoplan Monthly (N=71)
**Characteristic**									
**RORA**									
Mean (SD)	0.962 (0.508)	0.986 (0.502)	0.006 (0.474)	0.937 (0.479)	0.828 (0.513)	0.876 (0.483)	0.668 (0.779)	0.661 (0.854)	0.391 (0.499)
Median [Min, Max]	1.06 [0, 1.60]	1.05 [0, 1.60]	1.02 [0.0182, 1.60]	0.986 [0, 1.60]	0.763 [0, 1.60]	0.929 [0, 1.60]	0.434 [0, 3.46]	0.368 [0, 4.45]	0.185 [0, 2.19]
**PRD**									
Mean (SD)	1.30 (0.397)	1.34 (0.417)	1.41 (0.297)	1.29 (0.412)	1.25 (0.432)	1.29 (0.410)	1.75 (1.40)	1.83 (1.44)	1.40 (1.20)
Median [Min, Max]	1.51 [0.340, 1.62]	1.55 [0.0266, 1.60]	1.56 [0.116, 1.60]	1.46 [0, 1.60]	1.38 [0.0651, 1.61]	1.49 [0.0420, 1.60]	1.53 [0, 5.43]	1.39 [0, 5.17]	1.21 [0, 5.42]
**PRD in isolation**									
Mean (SD)	0.300 (0.263)	0.314 (0.259)	0.379 (0.314)	0.323 (0.237)	0.390 (0.291)	0.384 (0.272)	0.982 (0.891)	1.08 (0.924)	0.925 (0.803)
Median [Min, Max]	0.230 [0, 1,10]	0.217 [0, 896]	0.332 [0, 1.36]	0.318 [0, 1.07]	0.368 [0, 1.33]	0.343 [0, 1.23]	0.587 [0, 3.84]	0.829 [0, 3.83]	0.752 [0, 3.51]
**RPE loss**									
Mean (SD)	1.00 (0.518)	1.02 (0.510)	1.03 (0.468)	0.965 (0.483)	0.856 (0.517)	0.905 (0.492)	0.791 (0.870)	0.765 (0.896)	0.487 (0.568)
Median [Min, Max]	1.10 [0.00420, 1.60]	1.08 [0, 1.60]	1.14 [0.0252, 1.60]	0.994 [0, 1.60]	0.779 [0, 1.60]	0.944 [0, 1.60]	0.504 [0, 4.10]	0.441 [0, 4.46]	0.260 [0, 2.53]
**HTR**									
Mean (SD)	1.05 (0.483)	1.08 (0.484)	1.11 (0.433)	1.06 (0.481)	0.961 (0. 492)	1.00 (0.462)	0.772 (0.862)	0.853 (0.932)	0.534 (0.653)
Median [Min, Max]	1.19 [0.0665, 1.60]	1,25 [0.0168, 1.60]	1.24 [0.0336, 1.60]	1.15 [0, 1.60]	1.03 [0, 1.60]	1.10 [0, 1.60]	0.518 [0, 4.42]	0.518 [0, 4.50]	0.312 [0, 3.26]
**Intact macula**									
Mean (SD)	0.282 (0.394)	0.256 (0.417)	0.186 (0.297)	0.292 (0.381)	0.358 (0.431)	0.315 (0.410)	3.07 (1.59)	3.18 (1.52)	3.62 (1.27)
Median [Min, Max]	0.0756 [0, 1.26]	0.0420 [0, 1.57]	0.0308 [0, 1.48]	0.0980 [0, 1.58]	0.224 [0, 1.54]	0.109 [0, 1.56]	3.44 [0, 5.40]	3.66 [0, 5.27]	3.79 [0.00980, 5.35]

ETDRS region		7			8			9	
Treatment	Sham Combined (N=65)	Pegcetacoplan EOM (N=61)	Pegcetacoplan Monthly (N=71)	Sham Combined (N=65)	Pegcetacoplan EOM (N=61)	Pegcetacoplan Monthly (N=71)	Sham Combined (N=65)	Pegcetacoplan EOM (N=61)	Pegcetacoplan Monthly (N=71)
**Characteristic**									
**RORA**									
Mean (SD)	0.822 (0.923)	1.07 (1.09)	0.862 (1.02)	0.763 (1.07)	0.865 (0.957)	0.897 (0.925)	0.653 (0.963)	0.593 (0.755)	0.356 (0.530)
Median [Min, Max]	0.503 [0, 3.82]	0.776 [0, 4.02]	0.490 [0, 4.66]	0.265 [0, 3.85]	0.622 [0, 4.06]	0.772 [0, 3.47]	0.274 [0, 5.02]	0.287 [0, 3.39]	0.146 [0, 2.82]
**PRD**									
Mean (SD)	1.77 (1.44)	2.17 (1.64)	1.78 (1.40)	1.83 (1.65)	2.19 (1.65)	2.15 (1.49)	1.47 (1.44)	1.45 (1.36)	1.16 (1.05)
Median [Min, Max]	1.30 [0, 4.97]	2.02 [0, 5.15]	1.56 [0, 5.37]	1.30 [0. 5.37]	2.01 [0, 5.43]	1.89 [0, 5.12]	0.978 [0, 5.37]	1.15 [0, 5.29]	0.882 [0, 4.72]
**PRD in isolation**									
Mean (SD)	0.810 (0.658)	0.975 (0.816)	0.841 (0.666)	0.999 (1.00)	1.23 (1.14)	1.18 (0.900)	0.744 (0.698)	0.820 (0.847)	0.761 (0.751)
Median [Min, Max]	0.612 [0, 3.52]	0.787 [0, 3.29]	0.774 [0, 3.39]	0.590 [0, 4.87]	0.954 [0, 4.34]	1.00 [0, 3.78]	0.597 [0, 3.03]	0.619 [0, 4.91]	0.630 [0, 4.12]
**RPE loss**									
Mean (SD)	0.967 (1.01)	1.20 (1.18)	0.945 (1.06)	0.843 (1.12)	0.974 (1.00)	0.980 (0.969)	0.732 (1.03)	0.638 (0.781)	0.398 (0.549)
Median [Min, Max]	0.609 [0, 4.03]	0.878 [0, 4.36]	0.562 [0, 4.86]	0.314 [0, 413]	0.707 [0, 4.23]	0.780 [0, 3.78]	0.331 [0, 5.19]	0.339 [0.344]	0.225 [0, 2.88]
**HTR**									
Mean (SD)	0.920 (0.973)	1.25 (1.17)	1.01 (1.07)	0.950 (1.18)	1.12 (1.13)	1.12 (1.09)	0.788 (1.05)	0.740 (0.866)	0.494 (0.681)
Median [Min, Max]	0.601 [0, 4.31]	0.978 [0, 4.25]	0.636 [0, 4.67]	0.559 [0, 4.14]	0.798 [0, 4.16]	0.851 [0, 3.86]	0.274 [0, 5.13]	0.356 [0, 4.17]	0.259 [0, 4.05]
**Intact macula**									
Mean (SD)	3.25 (1.45)	2.89 (1.61)	3.21 (1.50)	2.91 (1.71)	2.62 (1.64)	2.72 (1.51)	3.37 (1.66)	3.57 (1.51)	3.84 (1.27)
Median [Min, Max]	3.51 [0, 5.24]	2.95 [0.183, 5.41]	3.30 [0.0406, 5.41]	3.09 [0, 5.43]	2.87 [0, 5.28]	2.82 [0, 5.43]	3.64 [0, 5.37]	3.82 [0.0420, 5.41]	4.05 [0.0882, 5.41]
